# Submerged macrophyte self-recovery potential behind restoration treatments: sources of failure

**DOI:** 10.3389/fpls.2024.1421448

**Published:** 2024-07-16

**Authors:** Michał Rybak, Joanna Rosińska, Łukasz Wejnerowski, Maria A. Rodrigo, Tomasz Joniak

**Affiliations:** ^1^ Department of Water Protection, Institute of Environmental Biology, Faculty of Biology, Adam Mickiewicz University, Poznań, Poland; ^2^ Department of Environmental Medicine, Poznan University of Medical Sciences, Poznań, Poland; ^3^ Department of Hydrobiology, Institute of Environmental Biology, Faculty of Biology, Adam Mickiewicz University, Poznań, Poland; ^4^ Integrative Ecology Group, Cavanilles Institute of Biodiversity and Evolutionary Biology, University of València, Paterna, Spain

**Keywords:** aquatic plant self-recovery, propagule bank, germination test, lakes eutrophication, lakes restoration

## Abstract

When exploring the challenges of restoring degraded lakes, we often do not observe the expected results despite executing all planned activities. Our study elucidates the reasons that impede the recovery of submerged macrophytes despite ameliorated light conditions. When prolonged lake degradation occurs, subsequent efforts to increase light availability often prove insufficient, resulting in a persistent turbid water state. In this study, we attempted to determine the reasons for these failures through a germination test and propagule bank analysis conducted in bottom sediments from a severely degraded lake, which underwent restoration. Although the bottom sediments indicate relative potential in the number of oospores and seeds, their germination efficacy remained dismally low. Based on the germination test results and factors affecting the development of submerged macrophytes (physical and chemical parameters, lake morphology), we stated that improvement of light conditions in the lake could be insufficient to recover the vegetation, especially when the potential to renew diverse plant communities from sediments naturally is low. Our findings advocate for a paradigmatic shift in lake restoration strategies. A holistic approach that includes propagule bank assessments before embarking on restoration initiatives and enabling the identification of macrophyte resurgence potentials is recommended. We also advocate for a multifaceted restoration framework, emphasizing the indispensability of augmenting natural recovery mechanisms with targeted interventions. Consequently, in some cases, macrophyte reintroduction could be the only solution. By reintroducing autochthonic species to site-specific ecological dynamics, we anticipate an increased success rate in restituting submerged vegetation, thus catalyzing ecological regeneration within degraded lake ecosystems.

## 1 Introduction

For several decades, eutrophication has constituted one of the threats caused by human activity; turbid water, cyanobacteria blooms, and loss of biodiversity are among the main symptoms visible in many lake ecosystems around the world that experience increased anthropogenic pressure (Sand-Jensen et al., 2017; Klimaszyk and Gołdyn, 2020). Lake eutrophication also leads to the disappearance of submerged plants and macroalgae (Hilt et al., 2006; Ozinga et al., 2009). As a result, the lake’s clear-water state with macrophytes dominance collapses, confronting high turbidity caused by phytoplankton or competition with free-floating plants (Scheffer et al., 1993; Meijer, 2000). Macrophytes play an essential role in the mobilization, transport, and accumulation of nutrients, and physical stabilization of bottom sediments. They are a source of food and refuge for macroinvertebrates, zooplankton, and fish (Short et al., 2016; Brzozowski et al., 2022). The limitation of macrophyte occurrence to a rush belt (helophytes) is a characteristic feature of degraded lakes (Kolada, 2016). The lack of bottom sediment insulation due to submerged plant disappearance intensifies the resuspension of sediments, and the biogenic substances are released from substrates into the water column, further accelerating eutrophication processes (Blindow et al., 2014).

Almost all undertaken restoration initiatives assume that reduction of nutrient concentrations in water is a crucial factor limiting phytoplankton abundance and, consequently, results in increased light penetration and the development of macrophytes, especially submerged communities. These organisms can compete effectively with phytoplankton and bring long-term improvement (Bakker et al., 2013; Hilt et al., 2018). Among physical factors, light is necessary for charophyte germination (de Winton et al., 2000), may enhance plant germination (Lorenzen et al., 2000) and is crucial factor for seedling development and submerged plant growth (van den Berg et al., 1998; He et al., 2019). When light conditions are improved, macrophytes can massively (re)establish and rapidly develop beds in the lake (van de Haterd and Ter Heerdt, 2007). The vegetation recovery can take from a few weeks to even a few years. There are many examples of lakes that have been restored, and the recovery of plants has been observed there (e.g., Lake Terra Nova or Lake Zwemlust, Netherlands; Lake Leven, United Kingdom; Lake Tegel, Germany; Bakker et al., 2013). Usually, the so-called ‘intermediate recovery state’ was noted, thus the period in which transient colonization by hornworts, pondweeds, waterweeds or charophytes occurred, especially in shallow lakes (Hilt et al., 2018). However, the clear-water state and return of submerged macrophytes and the development of their diversity are usually slow and often delayed. They can appear many years later, after limiting the external nutrient load and implementing complex restoration treatments (Bakker et al., 2013; Hilt et al., 2018).

However, sometimes improvement of light and trophic conditions is not followed by macrophyte re-establishment. One of the critical factors affecting the rate of bottom recolonization by macrophytes is the density and composition of the propagule bank (Bakker et al., 2013). There are two functionally different fractions in a lake propagule bank: i) an active fraction, capable of germinating under certain conditions (Hilt et al., 2006) and ii) a passive fraction, temporarily unable to germinate (dormant state, immaturity, isolation by a too thick sediment layer). The second fraction should be expected to dominate in degraded lakes with a permanent turbid water state. Nevertheless, seeds in the sediments suggest the possibility of natural regeneration of macrophyte communities once light conditions improve. Some studies have shown that seed banks from the most degraded lakes can also grow and have the potential to restore vegetation (de Winton et al., 2000; Verhofstad et al., 2017). However, the propagule bank in sediments of degraded lakes usually has a low number of viable seedlings. They exhibit strong seed dormancy and germinate in response to strict germination cues. Thus, the chances of recovering submerged vegetation by germination in such lakes are low (Rodrigo et al., 2013; Hilt et al., 2018).

Many studies focus on the reconstruction of submerged vegetation mainly in shallow lakes, while in deep lakes and reservoirs this aspect is neglected, since macrophytes colonize only a small part of the bottom. However, the recovery of submerged plants in deep lakes is as crucial as in shallow lakes because it also contributes to stabilization of ecological state in them (Hilt, 2015). Thus, there are still numerous knowledge gaps on propagule availability and dispersal for the return of submerged macrophyte vegetation during and after lake restoration treatments (Bakker et al., 2013; Hilt, 2015), and this problem is especially true in deep lakes.

The aim of our study was to assess whether meeting the basic assumption of all restoration projects, which is improving the light conditions in the lake, is sufficient for the self-recovery of macrophytes. To elaborate on a suitable basis, we performed a germination test and propagule bank analysis in sediments collected from a relatively deep lake that had been degraded for decades and had undergone restoration treatments. Such an approach gives a basic understanding of the conditions and structure of the propagule bank, and therefore its potential ability to germinate and the recovery of macrophytes. Based on the possible sources of failure, we proposed some actions that may increase possibility of macrophyte recovery. We emphasize that the described problems are underestimated and frequently overlooked. Awareness of the importance and relationships between predictor variables describing submerged aquatic vegetation coverage and composition can significantly facilitate and improve active protection and restoration efforts for aquatic vegetation recovery.

## 2 Methods

### 2.1 Study area

To check the possibilities of plant self-recovery, we collected sediments from Góreckie Lake (western Poland). This relatively deep stratified lake had been degraded for many years and had undergone restoration treatments to improve the quality of water. Additionally, it has been the focus of scientific interest for several decades, resulting in long-term research results and a well-studied vegetation structure (Dąmbska et al., 1978; Burchardt et al., 2001; Sobczyński et al., 2012; Rybak et al., 2015). Góreckie Lake (N 52°16’00”, E 16°47’43”) is endorheic, characterized by an area of 104.1 ha, max. depth of 17.2 m, and mean depth of 8.9 m. The originally mesotrophic lake underwent accelerated eutrophication from the 1950s to the 1980s due to the inflow of only mechanically treated wastewater from a sanatorium located on its shore, and the runoff from the agricultural catchment (Sobczyński and Joniak, 2009). As a result, the lake was hypereutrophic at the turn of the century (Sobczyński and Joniak, 2013) – 0.160 mg P L^-1^ total phosphorus (TP), 2.70 mg N L^-1^ total nitrogen (TN), and 1.1 m Secchi disk visibility (SDV). During our sampling time surface water was characterized by 0.046 mg P L^-1^ TP, 1.82 mg N L^-1^ TN, and 4.2 m SDV. Phytoplankton blooms were reported from early spring to late autumn, resulting in a substantial reduction in the extent of the euphotic zone and lack of oxygen in the meta- and hypolimnion (Sobczyński et al., 2012).

Due to high degradation and poor ecological state, restoration treatments were applied in April 2010 and were carried out with varying intensity in 2010 and 2012. To improve water transparency, oxygenate the bottom waters and reduce phosphorus concentration, three methods were applied simultaneously: aeration of hypolimnion waters using a wind-driven aerator, chemical phosphorus inactivation using iron sulphate salts, and biomanipulation by removing 3 tons of plankton-eating fish (Sobczyński et al., 2012). The goal of these activities was to support the natural defense of the lake, including the recovery of macrophytes as a factor regulating the circulation of biogenic elements in the ecosystem. However, the methods employed did not bring the expected effect on water quality improvement, and after they were discontinued, the lake returned to its pre-restoration condition. The main reasons for the low effectiveness of the treatments were the extensive hypolimnion and strong internal loading (Rybak et al., 2015; Joniak et al., 2018).

**Figure 1:**
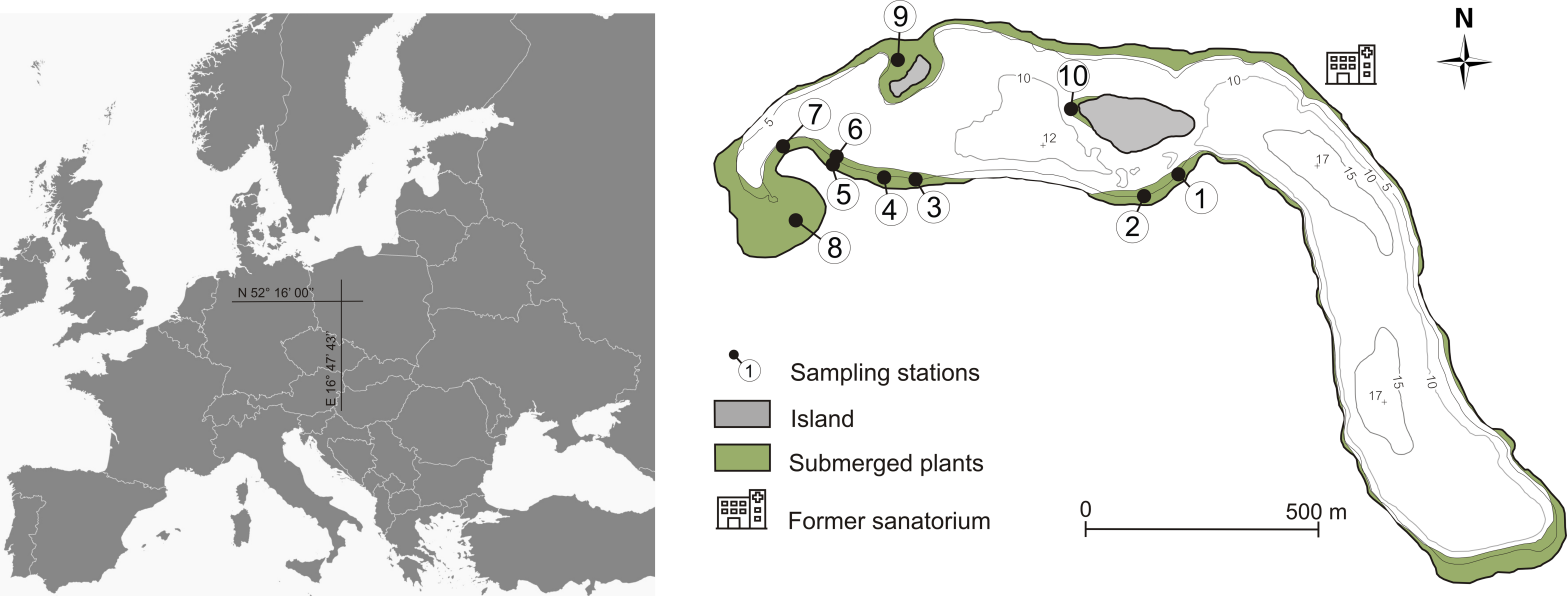
Bathymetric map of Góreckie Lake with sampling stations and surface area of the bottom covered by macrophytes in the 1952 – 1978 period.

In the early 20^th^ century, vegetation reached a maximum depth of 7m (**Figure 1**), *Chara tomentosa* and *C. globularis* were noted (Dąmbska, 1952). In the 1970s *Lychnothamus barbatus* occurred in the northern lake part (Dąmbska et al., 1978). By the early 21^st^ century, elodeids depth was 4 m in the northwest area of the lake, with *C. aspera* and *C. globularis* persisting (Burchardt et al., 2001). In 2013, six phytocoenoses (*Charetum contrariae*, *Myriophylletum spicati*, *Najadetum marinae*, *Nupharo-Nymphaeetum albae*, *Potametum pectinati*, *Potametum perfoliati*), indicating eutrophic waters, covered 7.7% of the phytolittoral zone.

### 2.2 Field measurement and sampling

Field studies and sampling were conducted in April (time before germination begins) 2013, one year after restoration treatments terminated. Sediment samples for the germination test were collected from 10 sites located in shallow lake parts (1.0–6.0 m), which were determined based on the submerged vegetation occurrence in the past (**Figure 1**; **Table 1**). Three sediment cores were collected from each site using the Kajak sampler. The samples at particular stations represented 8.47 × 10^-3^ m^2^ sediment surface and a volume of 1.27 L. The cores were then divided into three sediment subsamples based on the following strata: 0–5, 5–10 and, 10–15 cm and mixed within the layer. We cut the sediment cores into layers to assess the abundance and quality of the propagule bank. Then it was possible to determine whether propagules are present in a similar amount in the sediment along its entire depth or whether they are dominant in a particular layer (de Winton et al., 2000; Boedeltje et al., 2002). The sediments were stored in polypropylene containers at 4°C and analyzed within 24 hours.

**Table 1:** Density of diaspores in bottom sediments and physical characteristics of sampling stations of Góreckie Lake.

Station	Density of diaspores (thous. ind. m^-2^)		Depth of water(m)	Slope angle (°)	Width of the reed bed (m)	Thickness of fresh sediment (cm)	% PAR availability at the bottom	
	Oospore	Seed					spring	summer
1	12.31	1.54	6	8	16	5	1	0
2	3.59	0.51	6	11	14	15	1	0
3	16.92	2.05	4	9	14	3	4	1
4	14.87	0.51	4	9	14	3	4	1
5	160.00	5.64	2	10	14	3	16	8
6	8.72	0.51	5	7	14	3	2	0
7	38.46	2.05	3	12	16	2	9	2
8	30.26	lack	2	3	29	7	16	8
9	42.05	4.62	2	11	21	5	16	8
10	1.54	2.05	1	11	79	4	47	46

The following physical and chemical variables were analyzed in the sediment samples: total phosphorus (TP, mg P g^-1^ dry weight (dw)) by the molybdate method (PN-EN ISO 6878:2006) after mineralization with HCl, organic matter (OM, % dw) as loss on ignition at 550°C, calcium carbonates (CaCO_3_, % dw) using Scheibler method with HCl (Van Reeuwijk, 2002) and sediment hydration (Hydr, %) as fresh water lost on oven drying at 80°C for 48h.

Photosynthetically active radiation (PAR) was examined at 0.1 m intervals using a quantum meter with a spherical sensor (LI-193SA, LiCor Biosciences). Measurements were made in cloudless weather at the time of maximum solar height above the horizon. Additionally, measurements were repeated during the peak of vegetation season in July. The light extinction coefficient (K_d(PAR)_) was calculated according to Kirk (2010): $K_{d\left(PAR\right)}=\;\frac{lnI_{0}-\;lnI_{d}}{d}$ , where *d* is the water depth, *I_0_* and *I_d_* are PAR values at the water surface and at depth *d*, respectively. Data to measure the width of the reed belt and slope measurements (edge points and water depth) were collected with a Garmin Dakota 20 GPS and compiled using QGIS 3.12.

### 2.3 Germination test

We conducted the laboratory test using lake sediments based on the studies on germination potential, which have been assessed under different conditions (laboratory, greenhouse, outdoor) and on various substrates (e.g., natural sediments, sand, artificial substrate) (Thompson et al., 1997 and cited there). The germination test carried out during study employed methods used by de Winton et al. (2000) and Dugdale et al. (2001). It was performed in a cultivation chamber in glass cylinders. The cylinders were filled with filtered lake water (GF/F filters, Advantec) up to 25 cm of the water column height. The temperature was constant at 17.5°C, while the light regime was set at 10: 14 h (light: darkness). Light intensity at the water surface was 34.5 µmol m^-2^ s^-1^. This simulated lake conditions in the early spring period. Ninety transparent polypropylene plastic pots (120 cm^3^) were filled with 40 cm^3^ (2 cm height) of sediment subsamples from the particular layer. Each single pot was inserted in a single cylinder. Every layer from each station was replicated three times, which created a 0.165 m^2^ sediment area. Individuals of *Daphnia magna* L. from a long-term laboratory culture (Bdem2) were introduced in the cylinders to reduce the growth of phytoplankton (three per cylinder). Growths of filamentous algae were removed daily. The cultivation trial was carried out for 50 weeks.

The dissolved oxygen (HI 9146–04, Hanna Instruments), temperature, pH and electric conductivity (HI 98129, Hanna Instruments) were measured every two weeks. After 25 weeks, sediments were gently mixed to move potentially buried propagules closer to light. Emergence was defined as developing a germinated seedling to a stage where it could be identified. At the end of the test, each sediment sample was sieved by 250 µm aperture mesh, and the extract was observed using a stereoscopic microscope (Zeiss Stemi 2000-C) to identify and quantify the seeds and other propagules (e.g. charophyte oospores). Data of propagule numbers and seedlings were standardized to individuals per m^2^ (ind. m^-2^).

### 2.4 Statistical analyses

The differences in the number of propagules among sediments layers were analyzed with generalized linear mixed effect models from *glmmTMB* library (Brooks et al., 2017). Sampling station was incorporated as a random effect and the gamma family with log-link function was used to describe the data distribution. Non-parametric Kruskal-Wallis test (*stats* library) was used to check the differences of physicochemical features among sediment layers (TP, OM, Hydr, and CaCO_3_ content). Q-Q plot and residual plot were used to test the normality of the data.

The Principal Component Analysis (PCA) with factors grouping using k-means clustering (both in the *stats* library) was performed to visualize the data cloud, which allowed the identification of the distribution patterns of physical-chemical factors (water depth, fresh sediment depth, organic matter, hydration, total phosphorus, calcium carbonate, and availability of photosynthetic active radiation) and propagules and group them. The cluster number and its content was estimated using parallel analysis in the *paran* package (Dinno, 2018). Statistical analyses were performed using the R software (ver. 3.5; R Development Core Team).

## 3 Results

### 3.1 Environmental factors and sediment structure

The maximum PAR penetration depth in the lake was 10.5 m in spring. The percentage of the incident light at the bottom of the sampling stations ranged between 1% (12 µmol m^-2^ s^-1^) at 6 m and 47% (425 µmol m^-2^ s^-1^) at 1 m depth, with an average K_d(PAR)_ of 2.3 ± 1.0 m^-1^ (**Table 1**). All stations were in range of the euphotic zone where an average K_d(PAR)_ of 0.614 ± 0.092 m^-1^. At the peak of the growing season, the maximum light penetration depth was 5.5 m including euphotic zone depth of 3.3 m and an average K_d(PAR)_ = 1.449 ± 0.096 m^-1^. The light reaching the phytolittoral bottom was ca. 50% lower than in spring, and the K_d(PAR)_ increased to 4.8 ± 2.2 m^-1^.

The thickness of the fresh sediment layer ranged from 2 up to 15 cm (**Table 1**). In zones with the highest bottom slopes, the sediments contained a thin fresh layer. In areas with a lower slope, this layer was thicker. However, these differences did not significantly affect the analyzed physical and chemical features in the whole lake context (**Table 2**).

**Table 2:** Vertical changeability of physicochemical parameters (mean ± stand. dev.) of bottom sediments of Góreckie Lake (TP – total phosphorus, OM – organic matter, Hydr – hydration, CaCO_3_ – calcium carbonate; Z – Kruskal-Wallis test).

Parameter, unit	0–5 cm	5–10 cm	10–15 cm	Z	*p*	n
TP, mg P g^-1^ dw	10.1 ± 7.0	8.7 ± 7.6	6.0 ± 6.2	1.3	>0.05	30
OM, % dw	12.1 ± 13.3	9.0 ± 8.5	12.3 ± 10.7	0.4	>0.05	30
Hydr, %	66.0 ± 25.8	62.2 ± 16.9	68.2 ± 16.1	3.6	>0.05	30
CaCO_3_, % dw	16.3 ± 22.0	25.1 ± 22.7	26.7 ± 25.3	0.3	>0.05	30

**Figure 2:**
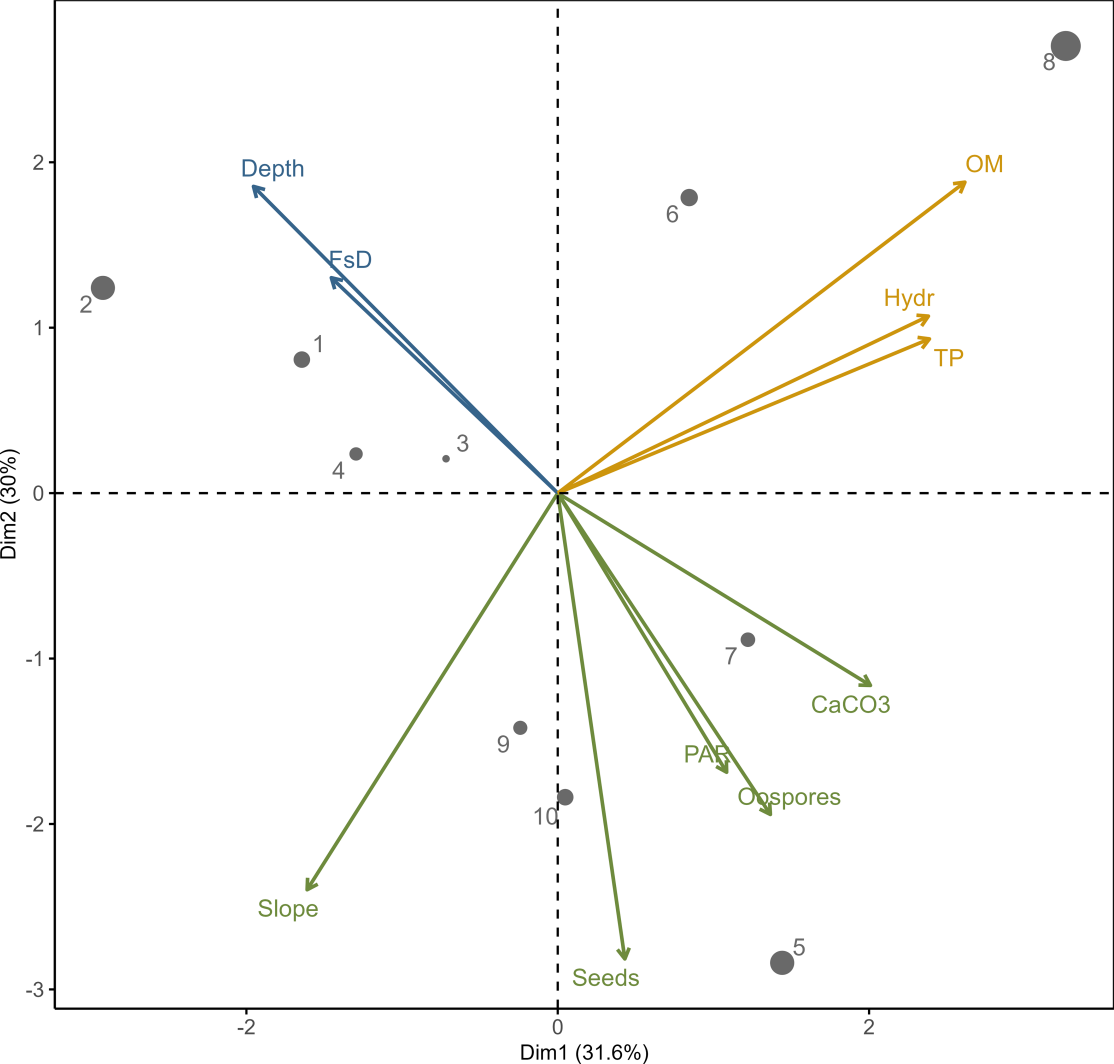
PCA output for the relationships between environmental variables with clustering algorithm results (denoted by color); depth – water depth, FsD – fresh sediment depth, OM – organic matter, Hydr – hydration, TP – total phosphorus, CaCO3 – calcium carbonate, PAR – availability of photosynthetic active radiation. Dots 1–10 – sampling stations according to **Figure 1** (the size indicates contribution of the variables to the principal components – up to 20%).

Variables describing sediments clearly indicated factors positively correlated with propagule bank abundance in the PCA ordination space defined by the first and second principal components (**Figure 2**). Both components accounted for more than 61% of the variance observed in the structure of the analyzed stations. The clustering algorithm revealed that environmental factors related to diaspore abundance could be divided into three groups. The first group includes factors positively correlated with propagule abundance: PAR availability, slope angle, and CaCO_3_ content. Factors in the second group were negatively correlated with propagule number, including water depth and fresh sediment layer thickness. The third group includes factors which were not correlated with propagules: sediment hydration, total phosphorus, and organic matter content.

### 3.2 Germination test

Water pH during the entire trial was constant and reached 8.5 ± 0.5. Dissolved oxygen in the first eight weeks was 6.5 ± 0.1 mg O_2_ L^-1^ and increased during the trial up to 9.7 ± 0.1 mg O_2_ L^-1^ in the last 4 weeks. Electric conductivity decreased from 800 ± 4 µS cm^-1^ in the first month to 640 ± 2 µS cm^-1^ at the end of assay.

Seedlings appeared after 6 weeks from station 8: *Chara virgata* Kütz. – 18 ind. m^-2^ from 0–5 cm and 36 ind. m^-2^ from layer 5–10 cm. After this time no new seedlings were noted nor did the sediment stirring induced emergence of new ones.

**Figure 3:**
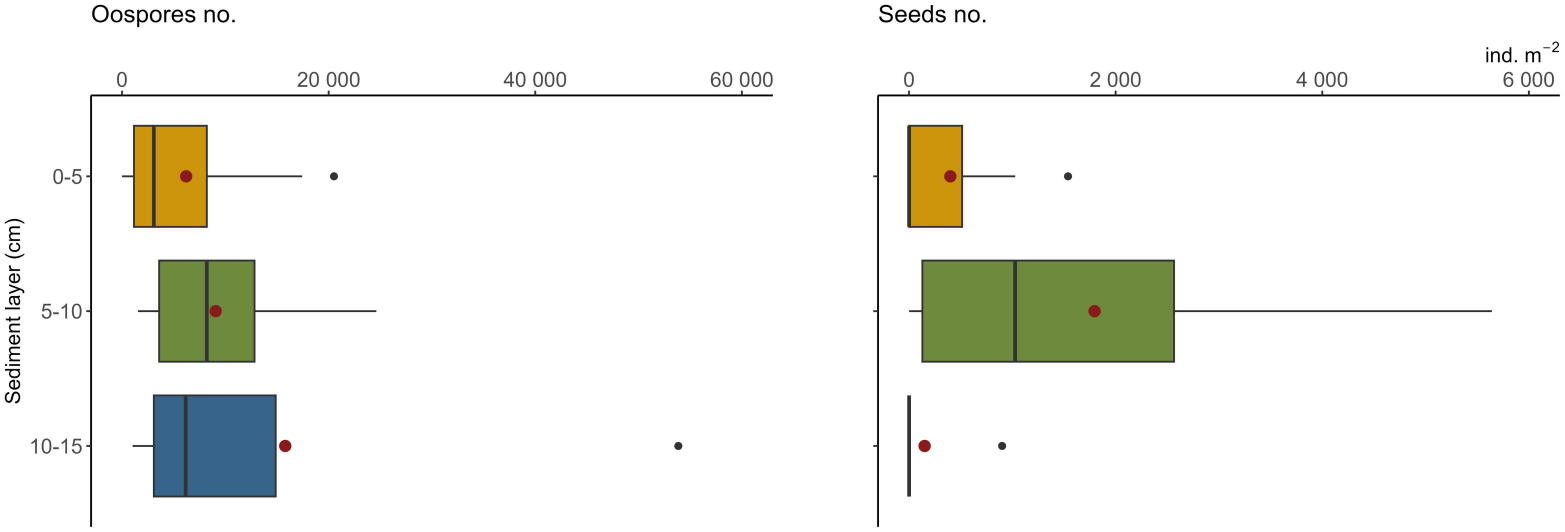
Densities of oospores (left) and seeds (right) in the bottom sediments used in the laboratory trial. For each layer 30 observations. Box-and whisker plots boxes represent median, first and third quartiles with whiskers extending until respectively the values that are within 1.5 × inter-quartile range; red dots represent the mean value, black dots represent outliers; oospores extreme of 160 thous. ind. m^-2^ not present (see **Table 1**).

Charophytes’ oospores were significantly more abundant than seeds in the propagule banks (F-ratio = 8.39, p<0.01) (**Table 1**). Considering the number of diaspores in particular layers, the highest number was noted in 5–10 cm (18 769 ± 9 960 oospores and 1 795 ± 702 seeds m^-2^; mean ± SE) and the lowest in 10–15 cm in the case of seeds (150 ± 150 ind. m^-2^) and 0–5 cm in the case of oospores (6 205 ± 2 317 ind. m^-2^). Statistical analysis did not reveal significant differences between sediment layers and sampling stations (**Figure 3**).

Microscopic analysis showed physical damage to about 80% of the seed pool (splitting, gnawing) and revealed that 70% of the oospores were empty carbonate encrustations with no biotic part.

## 4 Discussion

### 4.1 The propagules origin and the success of germination

Although we employed a standard methodology (de Winton et al., 2000; Dugdale et al., 2001) in the germination test conducted on sediments from Góreckie Lake, the results were significantly different from comparable studies. A key aspect that gives very good results in germination success, both in terms of germling density and plant taxonomic diversity, is the origin of the sediments. Ecosystems without signs of permanent degradation and aquatic vegetation occurrence are characterized by undisturbed production of diaspores (see e.g., Finlayson et al., 1990; Skoglund and Hytteborn, 1990; de Winton et al., 2000). If the lake has been degraded due to unnaturally fast eutrophication (such as Góreckie Lake), the inhibition of diaspore production and the replenishment of the propagule bank are the main problems (Lu et al., 2012). The density of propagules in Góreckie Lake ranged from ca. 500 (seeds) to 160 000 (oospores) ind. m^-2^, with the highest number in the 5–10 cm sediment layer. This proves the presence of a well-developed flora in the recent past. A relatively high number of diaspores, together with their potential survival (up to 50–60 years described by other authors; Lu et al., 2012; Rodrigo and Alonso-Guillén, 2013), seems to ensure the macrophytes return (Bakker et al., 2013). However, both the restoration treatments applied in the field and the laboratory germination test resulted in an infinitesimal success rate. A similar diaspore distribution pattern in the sediments was described in lakes where submerged plants were absent for years, however, viable seeds have been found (Hilt et al., 2006; Lu et al., 2012). The sediment analysis of Góreckie Lake demonstrated that the upper layer contained fewer seeds, which seems to be due to the very poor representation of elodeids in the lake, whereas this layer is crucial in terms of germination initiation potential. Seeds and oospores located deeper, even if numerous, exhibit lower germination potential and can only play an ecologically significant role in a situation of sediment disturbance. However, they must be disregarded when considering rapid vegetation regeneration (Dugdale et al., 2001; de Winton et al., 2004).

de Winton et al. (2000), collected 15 cm sediment cores divided into 5 cm layers; sediments were collected from lakes that supported seed production and lakes in which vegetation had disappeared (de-vegetated). The first seedlings were recorded after 5 weeks, and the highest number was found in the sediment of the 5–10 cm layer, however only in lakes with present vegetation. No seedlings were recorded from lakes classified as de-vegetated. These results are in line with our study, in which sediments origin from Góreckie Lake which could be classified as de-vegetated, considering submerged vegetation. Moreover, even mixing of the sediments in the germination test as described by van Zuidam et al. (2012), did not provide beneficial effects and did not contribute to seedling appearance.

Although the composition of the submerged propagule bank does not have to match the vegetation presence exactly (Arthaud et al., 2012), its condition indicates a new macrophyte emergence after a significant disturbance (Combroux et al., 2001). Thus, the spontaneous return of submerged macrophytes depends, at least partly, on the quality of the propagule bank. Viable propagules of non-dominant species, including charophytes, are usually found in most sediments. This means that when the dominant species disappears, there is a local potential for the development of diverse aquatic plants (Verhofstad et al., 2017), and species can be easily spread from neighboring ponds and water bodies (Barrat-Segretain, 1996). The data from lake sediments indicate that particularly propagules from Characeae and *Potamogeton* species are very abundant, and frequently appear in many restored lakes, as they can survive in turbid water (Bakker et al., 2013; Hilt et al., 2018). Corroborating this, the emergence of *C. virgata* seedlings from the oospores in Góreckie Lake sediments seems to be a result of dispersal by waterfowl (Vivian-Smith and Stiles, 1994; Linton and Goulder, 2000; Reynolds and Davies, 2007) from neighborhood ponds where *C. virgata* was present (Gąbka, 2009). In other lakes, e.g., Steinhuder Meer (Germany) the dominance of *Elodea nuttallii* was noted after a sudden improvement in water transparency approximately 40 years after the disappearance of plants. The species was not observed earlier in this lake and spread mainly by vegetative fragments (Hilt et al., 2006). Plants can also develop by expanding local remnant populations, as seen in Lake Fure, Denmark, where macrophytes spread through clones of several species from pre-eutrophication times (Bakker et al., 2013). If the dispersal inflow from external sources does not compensate a poor bank of propagules, the success in macrophyte establishment and development will gradually decrease (Van Onsem and Triest, 2018). As a result, passive fractions and the active propagule bank remains became critical bottlenecks for maintaining a clear-water state. In such a situation, the only way to restore vegetation in lake is artificial support of submerged macrophyte development through reintroduction of the most successful native species (Hilt et al., 2006; Rodrigo and Carabal, 2020).

### 4.2 Physical and chemical factors promoting successful germination

The submerged macrophyte distribution is regulated by a series of biotic and abiotic factors. Despite light availability (Cronin and Lodge, 2003) and nutrient concentration in both water and sediments (Feijóo et al., 2002; Li et al., 2007), water movement (Atapaththu and Asaeda, 2015), sediment anoxia (Zaman and Asaeda, 2013), and water column hypoxia (Summers et al., 2000) are pointed out as crucial. However, they always should be considered comprehensively. The failure of macrophyte recruitment suggests that the environmental conditions of Góreckie Lake were not beneficial for germination or the growth of submerged plants (Hay et al., 2008; Nonogaki, 2014). This is confirmed by the range of the euphotic zone in Góreckie Lake in spring and summer (10.5 and 5.5 m, respectively). Although the sediment illumination was sufficient, the submerged plant growth was strongly inhibited. Moreover, our germination potential test also exhibited negligible results. This underlines that many conditions must be met for successful germination. In many lakes, reducing nutrient concentrations and deepening the euphotic zone is sometimes not enough for renewal of macrophyte communities from the seed bank (Middelboe and Markager, 1997; Cronin and Lodge, 2003), as it is often assumed before restoration treatments.

The structure of the sediments in Góreckie Lake strictly reflected changes in trophic level. The organic layer was created mainly as a result of dead phytoplankton sedimentation, which developed substantial biomass during the vegetation season, and poor oxygen conditions in bottom waters prevented its decomposition (Rabalais et al., 2010; Sobczyński et al., 2012). Although low oxygen conditions might have positive effects on seed germination for some species (e.g. *Typha latifolia* and *Zostera marina;* Bonnewell et al., 1983; Moore et al., 1993), in general insufficient dissolved oxygen inhibits plant growth and intensifies other stressors, e.g., hydrogen sulfide (Parveen et al., 2017; Zhao et al., 2021). The mineral layer lying below referred to a higher calcium concentration, which was accumulated in sediments due to calcium carbonate precipitation on charophytes and macrophytes (Cicerone et al., 1999; Kufel et al., 2013; Pełechaty et al., 2013). The maximum sediment accumulation rate in the littoral zone was determined at 3.6 mm yr^-1^. This is a theoretical value on which many factors have an impact (Benoy and Kalff, 1999). Nevertheless, based on this rate, it can be assumed that the collected core sediments developed for ca. 40 years, and they date back to the period with well-developed macrophytes communities. Subsequently, accelerated eutrophication occurred leading to macrophyte disappearance and further lake degradation. On the other hand, the amount of the formed sediment can also be a factor that significantly influences germination. Seeds of some species (e.g. *Typha domingensis*) do not germinate when covered by sediment, however in contrast, others are able to grow through a thin layer of sediment or detritus (e.g. *Cladium jamaicense*) or could even withstand relatively thick sediments deposit, such as *M. spicatum* – up to 2 cm (Hartleb et al., 1993; Lorenzen et al., 2000), which was the sediment thickness in our germination test.

### 4.3 The importance of lake morphology in germination

The morphological, hydrographical, and catchment conditions of lakes may influence the trend and rate of theoretical changes in aquatic vegetation patterns. Since the macrophyte spatial distribution strongly depends on the water depth and light range, the maximum depth of a lake is one of the most critical factors determining the potential area covered by plants (Søndergaard et al., 2013). Consequently, propagule dispersal to the target habitat and successful germination in it are functional processes controlling plant community structure (Nilsson et al., 2010). It was demonstrated that a littoral slope over 2% caused a steep decrease in light availability and, in consequence, a rapid decrease in the vegetation distribution, abundance, and biomass (Duarte and Kalff, 1986; He et al., 2019). Differences in plant cover have also been shown between lakes with gentle slopes (shallow and deep regular-shaped) and ribbon-shaped lakes with steeper slopes (Kolada, 2014). This characteristic of the littoral area also determines the physical properties of sediments (nutrient poor) and the spatial differentiation of matter deposition, thus affecting the extent of vegetation. For example, sharp bottom slopes, which occur in Góreckie Lake, cause the top layer of sediments with propagules to slide down to the deeper zones of the lake and remove some parts of the propagules from the active seed bank. Thus, in lakes with a very steep bed slope, the abundance of macrophyte development may be naturally limited, even irrespective of water quality (Kolada, 2014). This was confirmed by the PCA analysis performed in our study, which showed that the number of diaspores in the sediment was related to the amount of light reaching it, which was inversely correlated with the depth and amount of fresh sediment (lake turbidity state phase). At the same time, it confirmed that the density of oospores was related to the amount of calcium carbonate intensively precipitated by charophytes and indicative of their abundant presence in the past. Correlating with the slope of the bottom, it indicates that diaspores may descend with the sediment to greater depths at the sharp slopes of the lake basin.

### 4.4 Broader perspective

Plant development was assumed to have a self-renewal characteristic due to the ecological state amelioration. However, these expectations may be illusory or may never come true without a proper propagule bank diagnosis and appropriate treatments to facilitate germination or macrophyte reintroduction (Rodrigo, 2021). The reduction of nutrient loading and biomanipulation (mainly fish removal) can bring a rapid response to improved light conditions and macrophyte development (Lauridsen et al., 2003). However, some treatments could also have a negative impact on plant recovery. For example, sediment removal may cause the removal of propagules or isolate them below the viable germination zone, and chemical phosphorus inactivation may inhibit charophyte generative reproduction (Hilt et al., 2006; Rybak et al., 2017, 2020). Climate changes should also be taken into account during restoration activities, because they affect plants, directly inducing morphological and physiological responses and indirectly interfering with biotic interactions, affecting the population dynamic (Tylianakis et al., 2008; Olsen et al., 2016). The stabilization of clear water phase by macrophytes might be limited in the hotter and carbon dioxide rich climate in the future. Considering climate change and the consequence of bloom-forming cyanobacteria domination in the phytoplankton community structure, only submerged vegetation with lower requirements for critical environmental factors can better withstand climate- and cyanobacteria-caused disturbances in the ecosystem. Surprisingly, charophytes seem to be good candidates as they can diminish the share of cyanobacteria in the lake phytoplankton more effectively than submerged vascular macrophytes and exhibit the potential to mitigate the effects of climate change (Pełechata et al., 2023). Therefore, future global warming effects should be considered in planning, management, and restoration since strategies may need to be refined and adapted to preserve or improve the present-day lake water quality (Trolle et al., 2011).

## 5 Conclusions and recommendations

We conclude that improving the light conditions in the lake may not be enough to recover vegetation, especially when the potential to naturally recover plant communities from sediments is low. We highlight that:

- Understanding the propagules germination-environmental factors-macrophytes occurrence interactions, offers a tool for improvement of the revegetation and management of many degraded lacustrine ecosystems.
- Analysis of the propagule bank before applying restoration treatments should be a standard procedure to recognize the possibility of macrophyte recovery from internal sources, particularly in lakes where no submerged plants are observed.
- The germination test should be done to assess the rate of germination success. Although many plants can reproduce clonally and spread without seed production, recruitment of seeds remains extremely important in the lake renewal or restoration context.
- If the oospores and seeds present in the propagule bank are not of high quality or viable, the only way to restore vegetation is artificial support of submerged macrophyte development through reintroduction of the most successful native species.

Such a strategy allows for better adjustment of restoration methods and saves time and resources in the event of the need for macrophyte reintroduction.

## Data availability statement

The raw data supporting the conclusions of this article will be made available by the authors, without undue reservation.

## Author contributions

MR: Conceptualization, Formal analysis, Investigation, Methodology, Resources, Visualization, Writing – original draft, Writing – review & editing. JR: Formal analysis, Investigation, Writing – original draft, Writing – review & editing. ŁW: Formal analysis, Investigation, Writing – original draft, Writing – review & editing. MAR: Formal analysis, Investigation, Writing – original draft. TJ: Conceptualization, Formal analysis, Investigation, Methodology, Resources, Validation, Writing – original draft, Writing – review & editing.
